# Lowering the density: ants associated with the myrmecophyte *Tillandsia caput-medusae* diminish the establishment of epiphytes

**DOI:** 10.1093/aobpla/plab024

**Published:** 2021-05-07

**Authors:** Carmen Agglael Vergara-Torres, Cecilia Díaz-Castelazo, Víctor Hugo Toledo-Hernández, Alejandro Flores-Palacios

**Affiliations:** 1 Departamento de Biología, División de Ciencias Biológicas y de la Salud, Universidad Autónoma Metropolitana, Iztapalapa, Ciudad de México 09340, México; 2 Red de Interacciones Multitróficas, Instituto de Ecología, A.C., Carretera antigua a Coatepec No. 351, El Haya, Xalapa, 91073, Veracruz, México; 3 Centro de Investigación en Biodiversidad y Conservación (CIByC), Universidad Autónoma del Estado de Morelos, Av. Universidad 1001, Col. Chamilpa, Cuernavaca, Morelos 62209, México

**Keywords:** Ant–plant interactions, plant establishment, plant–plant interactions, seed remotion

## Abstract

Ants benefit myrmecophytic plants by two main activities defending them from herbivores and offering nutrients. Ants’ territorial defence behaviour also benefits their myrmecophytic plants; in the case of trees, this behaviour includes eliminating structural parasites (epiphytes and lianas). These benefits could also occur with myrmecophytic epiphytes by decreasing the abundance of competing epiphytes. In two subunits of a tropical dry forest in the centre of Mexico, we (i) recorded the diversity of ants associated with the myrmecophyte *Tillandsia caput-medusae*, and experimentally tested: (ii) the effect of the ants associated with the myrmecophyte in the removal of its seeds and the seeds of other sympatric non-myrmecophyte species of *Tillandsia*; and (iii) if seed remotion by ants corresponds with epiphyte load in the preferred (*Bursera copallifera*) and limiting phorophyte species (*B*. *fagaroides*, *Ipomoea pauciflora* and *Sapium macrocarpum*). In five trees per species, we tied seed batches of *T. caput-medusae*, *T*. *hubertiana*, *T*. *schiedeana* and *T*. *recurvata*. One seed batch was close, and the other far away from a *T*. *caput-medusae* with active ants. Between forest subunits, ant richness was similar, but diversity and evenness differed. Ants diminish seed establishment of all the *Tillandsia* species; this effect is stronger in the forest subunit with a large ant diversity, maybe because of ant competition. Seed remotion by ants is independent of phorophyte species identity. Although ants can provide benefits to *T*. *caput-medusae*, they also could be lowering their abundance.

## Teaser

Ants provide several benefits to their myrmecophytic plants. In a tropical dry forest of Mexico, we experimentally found that ants associated with the myrmecophyte *Tillandsia caput-medusae* lower the density of seeds of all the *Tillandsia* species, including those of the myrmecophyte. This ant activity is harmful to the myrmecophyte and is more intense where ant diversity is higher, probably because competition between ants increases.

## Introduction

Ant–plant interactions are diverse in tropical forests. The ants can be antagonists (e.g. herbivores) but also can act as mutualists (e.g. diminishing herbivory, dispersing seeds, supplying nutrients), while plants can provide ants with food and nesting places (Hölldobler and Wilson 1990; Huxley and Cutler 1991; Jolivet 1996). In tropical forests, ants are the dominant arthropods (Floren *et al.* 2002; Watt *et al.* 2002) and can be up to 30 % of arthropods’ biomass in the canopy (Yanoviak and Kaspari 2000; Weiser *et al.* 2010).

Ants are highly efficient plant protectors because they have a predatory behaviour (eliminating or detreating herbivores). They establish a foraging territory that is cleaned, patrolled and protected from invaders (Huxley and Cutler 1991; Rico-Gray and Oliveira 2007). Several traits have been selected in plants associated with ants, including the offer of food resources for ants (e.g. extrafloral nectar) and structures for ant nesting (Heil and McKey 2003). However, ant–plant associations’ specificity is highly variable, ranging from obligate (e.g. the orchid *Coryanthes picturata* and the ant *Azteca gnava* live exclusively in ant-gardens; Morales-Linares *et al.* 2016) to facultative associations (e.g. the epiphyte *Epiphyllum phyllanthus* lives in or outside ant-gardens; Morales-Linares *et al.* 2016).

Myrmecophily is a facultative ant–plant association. In this association, the myrmecophytic plant provides ants with a nesting space. This space (domatia) is done by the plants inside special cavities in branches, trunks or between leaves (Hölldobler and Wilson 1990). Myrmecophytes benefit ants by providing them with nesting structures but also can offer food; in return, ants benefit myrmecophytes, protecting them from herbivores, providing nutrients inside the domatium and cleaning them from structural parasites (Janzen 1966; Beattie 1989; Rico-Gray and Thien 1989; Hölldobler and Wilson 1990; Davidson and McKey 1993; Treseder *et al.* 1995; Jolivet 1996).

**Figure 1.**
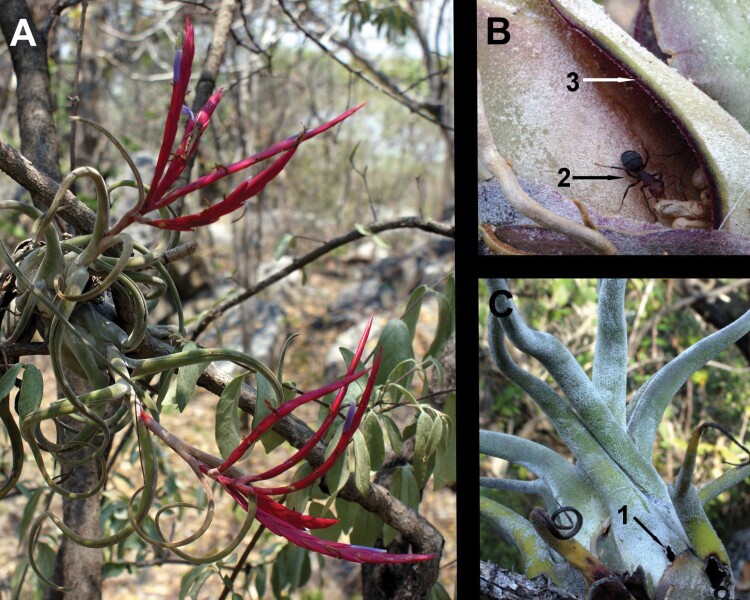
*Tillandsia caput-medusae*. We show in A: the plant with inflorescences. In B: an individual of *Camponotus rectangularis* (2) with its larvae and eggs, and the reddish inner layer of the cut leaf forming the domatia (3). In C: the entrance of a domatium (1).

In the Neotropics, at least 379 myrmecophytic plant species are known (including trees, shrubs, terrestrial herbs, lianas and epiphytes), represented by around 22 families (Chomicki 2019). Several epiphytic *Tillandsia* species of the Neotropical family Bromeliaceae are myrmecophytes (Davidson and Epstein 1989; Benzing 1990; Chomicki 2019). Myrmecophytic species of *Tillandsia* have the domatia between the widened leaf bases and ants do perforations to access inside these cavities (e.g. *Tillandsia butzii*, *T*. *bulbosa*, *T*. *caput-medusae*; Fig. 1; Benzing 1970).

Epiphytes are plants that, at least in one part of their life cycle, grow upon another plant (phorophyte) without contact with the forest floor and without developing parasitic structures (Flores-Palacios 2016). Epiphytes increase the heterogeneity of the forest and offer resources to the animals (Cruz-Angón and Greenberg 2005), increasing animal biodiversity (Dejean *et al.* 1992; Blüthgen *et al.* 2000; Volp and Lach 2019; Yanoviak *et al.* 2011), but also participate in the dynamics of the ecosystem, capturing water and nutrients from the atmosphere (Gotsch *et al.* 2016). Mirmecophytic epiphytes receive from the ants: nutrients and protection against herbivores (e.g. Rico-Gray and Oliveira 2007; Gegenbauer *et al.* 2012), but ants could also limit the establishment of competing epiphytes; as has been observed when ants eliminate structural parasites (vines, epiphytes) in myrmecophytic trees (Janzen 1966, 1967).

Comparatively, with terrestrial plant species, the studies about the effect of associated insects on epiphytes are few (Janzen 1966; Rico-Gray and Thien 1989; Dejean *et al.* 1992; Fisher 1992; Fiala *et al.* 1994; Yu 1994). In some Neotropical forest canopies, ants do not build carton nests and instead use myrmecophytes for nesting (e.g. Yanoviak *et al.* 2011; Vergara-Torres *et al.* 2018). In some tropical dry forests, myrmecophytes’ distribution is biased towards a few preferred phorophyte species (e.g. Vergara-Torres *et al.* 2010). In addition, it has been found that in phorophytes with a high epiphyte load, ant activity and seed remotion are high (Vergara-Torres *et al.* 2018). Therefore, the presence of myrmecophytic *Tillandsia* species could have a negative effect on the populations of epiphytes because the ants associated with myrmecophytes could be lowering seed establishment.

The tropical dry forest is a highly variable ecosystem, where plant and ant composition change within small spatial scales (Vergara-Torres *et al.* 2010, 2017; Trejo and Dirzo 2002). For example, in the tropical dry forest of San Andrés de la Cal, Tepoztlán, Mexico, there are two well-defined forest subunits, where ant activity and composition differ (Vergara-Torres *et al.* 2017) and could influence ant–plant interactions (e.g. increasing ant competition where ant abundance is higher; Vergara-Torres *et al.* 2018).

In this work, we experimentally tested whether the ants associated with the myrmecophyte *T. caput*-*medusae* removed its seeds and the seeds of other sympatric non-myrmecophyte *Tillandsia* species, diminishing the establishment of *Tillandsia* species. In two tropical dry forest subunits (lava-flow and limestone) where ant diversity and activity differ (Vergara-Torres *et al.* 2017, 2018), we determined the ants associated with *T*. *caput-medusae*, the plant traits (number of rosettes, average height and maturity of each rosette) that determine the presence of ants, and we perform an experiment of seed remotion. We hypothesized that: (i) ant richness associated with *T*. *caput-medusae* should differ between forest subunits (previous work shows that ant composition differs in 44 % between forest subunits; Vergara-Torres *et al.* 2017); (ii) ant will be associated with the bigger flowering plants (because these have the reproductive size and offer nectar in their inflorescences); (iii) the protection behaviour of the ants increases when the seeds fall close to the nest (*T*. *caput-medusae*); (iv) the remotion will be greater in phorophyte species with greater epiphyte loads, and at the lava forest subunit (according to previous findings; Vergara-Torres *et al.* 2018).

## Methods

### Study area

The study was done in the tropical dry forest of San Andrés de la Cal, Tepoztlán, Morelos, Mexico (18°57′22.2″N to 99°06′50.2″W, altitude 1480–1670 m a.s.l.). In this area, the climate is warm–subhumid, with a mean annual temperature of 18 °C and mean annual precipitation of 1000 mm (Comisión Nacional del Agua, unpubl. data; Vergara-Torres *et al.* 2010, 2017, 2018). Two subunits of tropical dry forest can be recognized in this area: one subunit develops in limestone rock and the other in lava-rock (Vergara-Torres *et al.* 2017, 2018). For each subunit, the communities of trees, epiphytes, ants and the epiphyte–phorophyte relationships are known (Vergara-Torres *et al.* 2010, 2017; Cortés-Anzúres 2015). Among forest subunits, tree composition differs **[see**Supporting Information**—Table S1****]**, but in both, the dominant tree species is the endemic endangered species *Sapium macrocarpum* (Euphorbiaceae; **see****Supporting Information—Table S1**). Nine epiphytic Bromeliaceae species are known in the limestone forest subunit and eight in the lava-rock subunit **[see****Supporting Information—Table S2****]**; in both subunits, *T. caput*-*medusae* comprises <3 % of the individuals.

Twenty-seven ant species are known in the study zone (Vergara-Torres *et al.* 2017), 19 species in the limestone forest subunit and 17 in the lava-rock subunit; nine species are shared between forest subunits. Fifty-six per cent of the ants are arboreal species, and the rest are habitat generalists (Vergara-Torres *et al.* 2017).

In both forest subunits, epiphytes’ distribution is biased between the phorophytes (Vergara-Torres *et al.* 2010; Cortés-Anzúres 2015). In the limestone forest subunit, three phorophyte species are preferred (*Bursera copallifera*, *B*. *glabrifolia* and *B*. *bipinnata*) while another five are limiting (*Conzattia multiflora*, *Ipomoea murucoides* and *I*. *pauciflora*, *Lysiloma acapulcense* and *S. macrocarpum*) (Vergara-Torres *et al.* 2010). In the lava-rock forest subunit, two phorophytes are preferred (*B*. *bipinnata* and *Quercus obtusata*) and four limiting (*S*. *macrocarpum*, *I*. *pauciflora*, *Salvia sessei* and *I*. *murucoides*) (Cortés-Anzúres 2015).

#### *Ants associated with T. caput*-*medusae.*

Fieldwork was done during February–September of 2015. To know the ant species associated with *T*. *caput-medusae*, we directly search in a sample of 107 individuals (57 in the limestone and 50 in the lava-rock forest subunit). The surveyed *T*. *caput-medusae* were in a 500 m path in each zone; each plant was tagged (aluminium tags), and we counted its number of rosettes. For each rosette, we measured its height (from the base to the longest leaf; López-Villalobos *et al.* 2008), and we noted if it was young (without inflorescences), a reproductive adult (with an active inflorescence) or senescent (the rosette was wilting and sustained a wilting/dry inflorescence). In each rosette, ant presence was noted moving the outer leaves; if ants came out, a sample was taken for identification. Ants were collected and preserved in 70 % ethylic alcohol (see Vergara-Torres *et al.* 2017). In the Insect Collection of the Autonomous University of Morelos State (CIUM), ants were identified and deposited.

#### Seed remotion experiment.

Before seed dispersion (March–April) occurred, mature fruits of *T. caput-medusae*, *T*. *hubertiana*, *T*. *schiedeana* and *T*. *recurvata* were collected (February–March 2015); since collecting the fruits in this period warrant the presence of mature seeds with >90 % germination (Flores-Palacios *et al.* 2015). Fruits were taken to the Ecology Laboratory (CIβγC), where they open in an oven at 30 °C (Binder, Model FD 115-UL). Thirty seeds per species were inserted into a cotton thread, leaving 1 cm between seeds. For each *Tillandsia* species, 40 seed batches were done.

We selected five individuals of the phorophytes: *B. copallifera* (preferred), *B*. *fagaroides*, *I*. *pauciflora* and *S*. *macrocarpum* (limiting). In each tree, we selected one branch with a *T*. *caput-medusae* with an active ant nest (i.e. we observed active ants emerging from the domatium, and we gently open some leaves to observe eggs, larvae or pupal), and two threads with seed batches per *Tillandsia* species were tied around this branch (2 batches × 4 *Tillandsia* species = 8 seed batches per branch). One seed batch was tied close to the *T*. *caput-medusae* (13.8 ± 10.6 cm; hereafter, we report mean ± SD) and the other far away (72.8 ± 30.1 cm). Each week we counted the number of seeds removed, germinated and not removed in each seed batch.

### Data analysis

In each forest subunit, we describe the community of ants associated with *T. caput-medusae* with three diversity measures, the species richness (^0^*D* true diversity; Jost 2010), the Shannon diversity index (^1^*D* true diversity, ^1^*D* = e^*H*′^) and the Pielou index of diversity [*H′*/ln(species richness)]. These indexes describe the communities in terms of the maximum possible true species diversity (^0^*D*), the expected number of species with the same abundance (equivalent species, ^1^*D*, Jost 2010) and the proportion of the maximum diversity observed in each community (Pielou evenness; Jost 2010). In the results we show the values of *H*′ and ^1^*D* for comparison with other works.

With a generalized linear model for binomial response variables and a logit link function (Crawley 1993), we tested which *T*. *caput-medusae* trait (number of rosettes and mean height of each type of rosettes) relates with the presence of ants.

Generalized linear models for binomial response variables (Crawley 1993) were used to test whether each *Tillandsia* species’ seed remotion depends on the forest subunit, phorophyte species and distance from the nest (the focal *T*. *caput-medusae*). For these models, the factors were: forest subunit and phorophyte (nested in forest subunit), while distance was a covariable. All analyses were done in R 3.6.3 (R Core Team 2020), with the libraries ggplot2 (graphs; Wickham 2016) and multcomp (multiple comparisons and contrast method; Bretz *et al.* 2010).

## Results

In total, nine ant species were found in *T*. *caput-medusae* (Table 1). In the limestone forest subunit, 43.3 % of the plants had ants, and 28.0 % in the lava-rock forest. *Camponotus rectangularis willowsi* (Fig. 1) was the most frequent species (Table 1), but in the lava-rock forest *Crematogaster curvispinosa* was almost as frequent as *C*. *rectangularis willowsi* (Table 1). In each forest subunit, we found a similar number of ant species (Table 1); however, the ant community associated with *T*. *caput-medusae* was 25 % most diverse in the lava-rock forest subunit (according to the ^1^*D* true diversity) and 33 % more even (according to the evenness Pielou index; Table 1).

**Table 1.** Ant diversity and percentage of ant species occurrence in plants of *T. caput-medusae* of two subunits (limestone and lava-rock) of a tropical dry forests in San Andrés de la Cal, Tepoztlán, Mexico.

Ant species	Forest subunit	
	Lava-rock (*n* = 50)	Limestone (*n* = 57)
Formicinae		
*Camponotus conspicuus zonatus*	2.0 %	3.5 %
*Camponotus mina*	–	1.8 %
*Camponotus rectangularis willowsi*	14.0 %	36.8 %
Myrmicinae		
*Cephalotes setulifer*	–	1.8 %
*Crematogaster curvispinosa*	12.0 %	–
*Nesomyrmex echinatinodis*	–	7.0 %
*Temnothorax* sp.	2.0 %	–
Pseudomyrmecinae		
*Pseudomyrmex pallidus*	–	1.8 %
*Pseudomyrmex subater*	2.0 %	–
Species richness or ^0^*D* =	5	6
*H*′ (^1^*D*) =	1.25 (3.5)	1.05 (2.8)
Pielou evenness, *J* =	0.78	0.58

The plants of *T*. *caput-medusae* were similar in size between forest subunits, but the young rosettes were 19 % larger in the lava-rock subunit (Table 2). In the limestone forest subunit, ants were more frequent (34.1 %) in adult rosettes, while in the lava-rock forest ants were more frequent (22.7 %) in young rosettes. The variables that were significantly related with ant presence were the heights of young (χ ^2^ = 8.8, *P* < 0.05) and of adult (χ ^2^ = 16.9, *P* < 0.05) rosettes, none other variable was related with ant presence (all χ ^2^ < 3.0, *P* > 0.05).

**Table 2.** Means (± SD) of the height and number of rosettes of *T*. *caput*-*medusae* plants found in two subunits (limestone and lava-rock) of tropical dry forests in San Andrés de la Cal, Tepoztlán, Mexico. In the last column we show the test statistic (*U*) for the Mann–Whitney test comparing each variable between forest subunits and its probability (*P*).

Trait	Lava-rock (*n* = 50)	Limestone (*n* = 57)	*U*	*P*
Number of rosettes	1.8 ± 1.3	2.2 ± 1.9	1321.5	0.51
Number of rosettes with ants	0.3 ± 0.6	0.5 ± 0.6	1196.0	0.15
Number of young rosettes	1.0 ± 0.8	1.1 ± 1.0	1377.5	0.77
Number of adult rosettes	0.5 ± 0.7	0.5 ± 0.7	1363.0	0.70
Number of senescent rosettes	0.4 ± 0.8	0.6 ± 1.0	1260.0	0.30
Height of young rosettes (cm)	20.9 ± 8.2	17.5 ± 8.9	603.5	<0.05
Height of adult rosettes (cm)	33.5 ± 6.8	34.9 ± 6.8	201.5	0.67
Height of senescent rosettes (cm)	26.8 ± 7.8	30.9 ± 5.1	90.5	0.25

**Figure 2.**
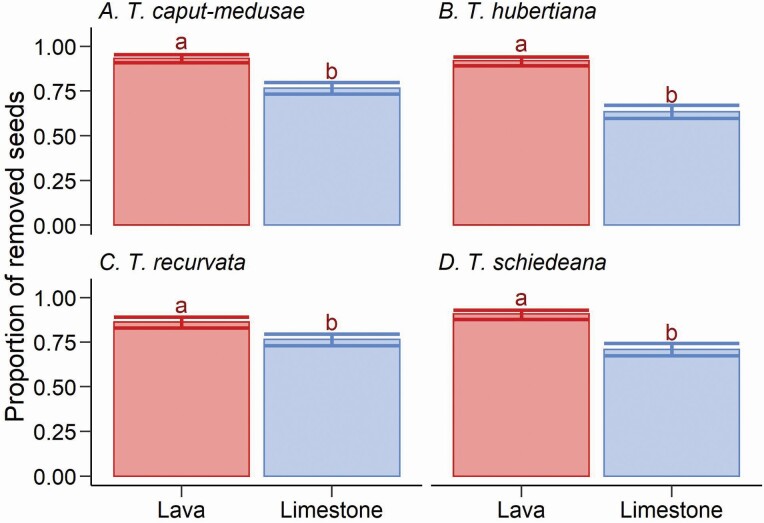
Seed remotion of four *Tillandsia* species (A, B, C, and D) in tropical dry forest developed on limestone (Limestone) and lava-rock (Lava). Lines of dispersion are the 95 % confidence interval for binomial variables. Inside each graph, different letters indicate a significant difference between the proportions.

**Figure 3.**
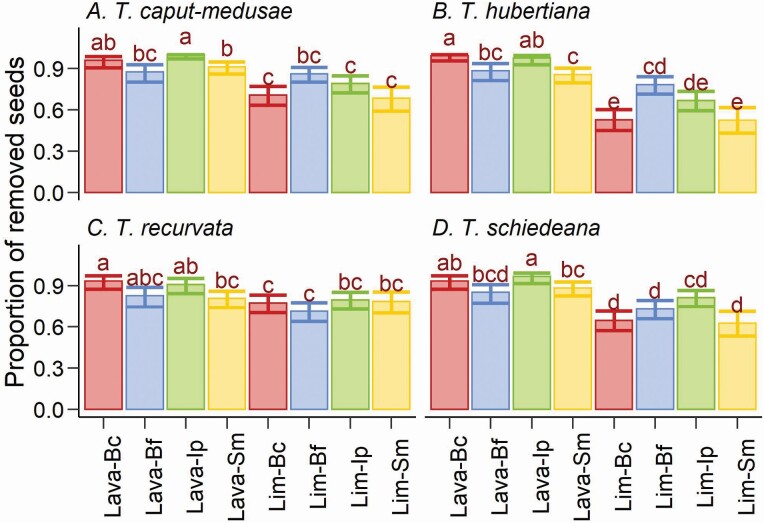
Seed remotion of four species of *Tillandsia* (A, B, C, and D) in the phorophytes *Bursera copallifera* (Bc), *B*. *fagaroides* (Bf), *Ipomoea pauciflora* (Ip) and *Sapium macrocarpum* (Sm) in tropical dry forest developed on limestone (Lim) and lava-rock (Lava). Lines of dispersion are the 95 % confidence interval for binomial variables. Inside each graph, different letters indicate a significant difference between the proportions.

For all the *Tillandsia* species, the remotion of seeds differed between forest subunits (Table 3). In all *Tillandsia* species, seed remotion was greater in the lava-rock forest subunit (Fig. 2). We found a significant effect of phorophyte species on seed remotion (Table 3); however, this effect was not caused by consisted differences between the phorophyte species; it was caused by differences between forest subunits (Fig. 3); only for *B. copallifera*, the remotion was greater when in the lava-rock forest subunit (Fig. 3). For the rest of the phorophytes species, seed remotion was not always greater when in the lava-rock subunit.

**Table 3.** χ ^2^ values indicating the effect of forest subunit, phorophyte species (nested in the forest subunit) and the distance to the myrmecophyte *Tillandsia caput-medusae* on the remotion of seeds of four *Tillandsia* species. ns = non significant, *P < 0.05, **P < 0.001, ***P < 0.0001.

Source of variation	*Tillandsia caput-medusae*	*Tillandsia hubertiana*	*Tillandsia recurvata*	*Tillandsia schiedeana*
Forest subunit	69.9***	144.3***	18.6***	75.6***
Phorophyte (forest subunit)	43.0***	64.2***	18.2**	30.3***
Distance	0.2^ns^	8.2*	1.8^ns^	11.3**

The effect of distance from *T. caput*-*medusae* on seed remotion was only significant for the seeds of *T*. *hubertiana* and *T*. *schiedeana* (Table 3); in these species, the effect of nest distance on seed remotion was weak and could be positive (*T*. *schiedena*, coefficient = 0.00108) and negative (*T*. *hubertiana*, coefficient = −0.005001).

## Discussion

Ants can be the dominant insects in the canopy (Davidson *et al.* 2003), and they establish mutualistic interactions with myrmecophytic epiphytes and trees (myrmecophily; Rico-Gray and Thien 1989). In myrmecophytic trees, ants remove seeds of structural parasites (Janzen 1967, 1969); we tested if this activity also occurs in the surroundings of the myrmecophytic epiphyte: *T. caput-medusae*.

We found a community of nine ant species occupied between 30 and 50 % of the *T*. *caput-medusae* plants. These values are like those found in a tropical dry forest of Quintana Roo, south of Mexico, where 56 % of the *Tillandsia bulbosa* plants (*n* = 42) were occupied by six ant species (Dejean *et al.* 1995). And also, as those found in the epiphyte orchid *Caularthron bilamellatum* in a tropical moist forest, where between 25 and 30 % of the plants (*n* = 542) were occupied by the two dominant ant species (Yanoviak *et al.* 2011). In addition, we observed one ant change during the review of the plants, from *Camponotus rectangularis* to *Nesomyrmex echinatinodis*. This change could be the result of ant competition for the use of the best *T*. *caput-medusae* domatia. In *Clidemia heterophylla* (Melastomataceae), ant species turnover in the domatia suggests ant competition (Davidson and Epstein 1989). The percentage of plant occupation by ants (≤50 %) indicates that the best rosettes could limit the ants. In *C. bilamellatum*, 58 % of all the available domatia were unsuitable for the ants (Yanoviak *et al.* 2011).

Ant presence was related with large young and large adult rosettes of *T*. *caput-medusae*, suggesting that ants search for large domatia, where some other resources will be available (e.g. nectar), and that the availability of these suitable domatia limits the ants (Volp and Lanch 2019). *Tillandsia caput-medusae* is a semelparous plant, each rosette grows until flowering/seed dispersion and then dies, but old wilting rosettes produce new rosettes before dying. The large young rosettes may be colonized from ant colonies that inhabited old decaying rosettes; thus, an ant colony can maintain its territory by recolonizing the same plant. Janzen (1966) observed that ant colonies of *Vachellia* trees moved up to three times between hollow thorns (domatia) during a single year. It is necessary to research which plant traits signal the ants to abandon or to colonize a domatium. We observed that active nests were inside domatium where the leaves have reddish inner surfaces (Fig. 1); while, the inner surface in small rosettes without ants is green, and in senescent rosettes is brownish. The reddish may be related to anatomical structures that help *T*. *caput-medusae* absorb nutrients while signalling the ants that the rosette will offer nectar during the next flowering season.

Our evidence confirms that the ants nesting in *T. caput*-*medusae* removes seeds of *Tillandsia* species. In a previous experiment (Vergara-Torres *et al.* 2018), the mean seed remotion was 24 % (reaching a maximum of 58 %); however, we did not warrant the presence of *T*. *caput-medusae* in the phorophytes. In the present experiment, we follow seed remotion in the surroundings of *T. caput-medusae* with nesting ants, and the general mean seed remotion was 81 % (with a maximum of 100 %). This evidence clearly shows that ants are cleaning seeds in their nest’s surroundings, but contrary to our expectations, this cleaning behaviour mainly depends on the forest subunit.

The main difference between forest subunit is the ant composition and diversity. In the limestone forest subunit, the diversity and evenness of the ant community nesting in *T*. *caput-medusae* were lower, and *C. rectangularis* was the most frequent species. In comparison, in the lava-rock forest subunit, the diversity and evenness were higher, and the most frequent species were *C. rectangularis* and *C. curvispinosa*. Arboreal ants of the genus *Crematogaster* include species that act as generalist predators and scavengers; among them, *C. curvispinosa* can be usually dominant and aggressively defend its territory, but there are reports that *C*. *curvispinosa* can also live in the same plant with species of the genera *Camponotus* or *Dolichoderus* (Longino 2003). It is possible that the higher seed remotion in the lava-rock forest subunit occurs because of significant competition between ants; as a consequence, ants in this forest subunit invest more time patrolling and cleaning their territories.

Contrary to our hypotheses, we did not find a pattern concerning the effect of phorophyte identity and distance from the nest in seed remotion. We found weak support for this hypothesis in *B. copallifera* only (a phorophyte with high epiphyte load); in each forest subunit, this phorophyte was always among those with significant seed remotion. For the rest of the phorophyte species, the seed remotion was not higher in the phorophytes with greater epiphyte loads and ant activity (Vergara-Torres *et al.* 2017, 2018).

Seed remotion was not affected by the distance to the nest in *T. caput*-*medusae* and *T*. *recurvata*, while in *T*. *hubertiana* and *T*. *schiedeana* the effect was weak, and only in *T. hubertiana* the outcome was as expected in the hypothesis. It has been found that ants patrol and disperse seeds in territories more extensive than the distance we assayed (Gómez and Espadaler 2013). It is possible that ants patrol and forage (e.g. visiting flowers in the upper twigs) in the entire tree where we performed our experiment, lowering our ability to found a remotion by distance pattern.

Ants can recognize their host myrmecophytes and their seeds by different chemical clues (Morales-Linares *et al.* 2018; Nelson *et al.* 2019). For example, in a tropical rain forest of the south of Mexico, individuals of *A. gnava* discriminate between the tiny seeds of an orchid associated with their ant-gardens and seeds of an orchid not present in their ant-gardens (Morales-Linares *et al.* 2018). We found that the ants associated with *T. caput-medusae* do not discriminate among the *Tillandsia* species’ seeds and remove all seeds, as had been suggested previously for epiphytes (Nelson *et al.* 2019). The overall mean seed remotion by species (the average between forest subunits) followed the descending gradient: *T*. *caput-medusae* (84 %, 95 % confidence interval = 82–86 %), *T*. *recurvata* (81 %, 95 % confidence interval = 78–83 %), *T*. *schiedeana* (80 %, 95 % confidence interval = 77–82 %) and *T*. *hubertiana* (76 %, 95 % confidence interval = 74–79 %). This generalist behaviour could explain the low abundance of *T*. *caput-medusae* in the tropical dry forest studied. *Tillandsia caput-medusae* produces many seeds (106 seeds per fruit), which have high viability and longevity (Flores-Palacios *et al.* 2015); still, this species is less abundant than other sympatric epiphytes and comprises <3 % of the epiphytic Bromeliaceae in the studied area. Seed remotion by ants reduces the establishment of new plants and could reduce their population size; this effect must be stronger in *T*. *caput-medusae*, because, in wind-dispersed species, most seeds fall near the mother plant. In contrast, other myrmecophyte species have seeds with rewards for the ants (e.g. elaiosome), those of *Tillandsia* lack rewards that promote the dispersion of the seeds by the ants.

## Conclusions

Our data support the notion that ants clean the surroundings of the *T*. *caput-medusae* where they nest, and this activity lowers the abundance of epiphytes. While the most abundant non-myrmecophyte epiphytes could tolerate this activity by a mass effect, it could be detrimental for *T*. *caput-medusae* (which have a low abundance). Lowering the abundance of *T*. *caput-medusae* is a negative effect of the ants on *T*. *caput-medusae*. Still, other aspects of the interaction must be researched, as the ants’ effect on the growth and reproduction of *T*. *caput-medusae*.

## Supporting Information

The following additional information is available in the online version of this article—

**Table S1.** Tree species abundance (Diameter at breast height > 3 cm) in two subunits of tropical dry forest in San Andrés de la Cal, Tepoztlán, Mexico. We show the percentage of individuals per species (Vergara-Torres *et al.* 2010; Cortés-Anzures 2015).

**Table S2.** Bromeliaceae species abundance in two subunits of tropical dry forest in San Andrés de la Cal, Tepoztlán, Mexico. We show the percentage of individuals per species (Vergara-Torres *et al.* 2010; Cortés-Anzures 2015).

plab024_suppl_Supplementary_Materials

## Data Availability

All data are available in files provided in the **Supporting Information** section.

## Notes

**Populations & Communities. Chief Editor:** Jean Burns
