# Supplementary material for: Lowering the density: ants associated with the myrmecophyte Tillandsia caput-medusae diminish the establishment of epiphytes
Source: AoB Plants. 2021 May 7;13(4):plab024. doi: 10.1093/aobpla/plab024 (PMC8266655; doi:10.1093/aobpla/plab024)
Supplement: plab024_suppl_Supplementary_Materials [file plab024_suppl_supplementary_materials.zip › plab024_suppl_Supplementary_Materials.docx]

Supplementary material

**Table S1**. Tree species abundance (DBH > 3 cm) in two subunits of tropical dry forest in San Andrés de la Cal, Tepoztlán, Mexico. We show the percentage of individuals per species (Vergara-Torres *et al.* 2010, Cortés-Anzures 2015).

| **Species** | **Forest subunit** | | |
| --- | --- | --- | --- |
|  | **Lava-rock** | **Limestone-rock** |  |
| **Apocynaceae** |  |  |  |
| *Plumeria rubra* L. | 3.0% | 0.7% |  |
| *Thevetia thevetioides* (Kunth) K. Schum. | 1.3% | 2.6% |  |
| **Burseraceae** |  |  |  |
| *Bursera bicolor* (Willd. ex Schltdl.) Engl. | -- | 1.4% |  |
| *B. bipinnata* (DC.) Engl. | 2.6% | 2.4% |  |
| *B. copallifera* (DC.) Bullock | 1.3% | 2.6% |  |
| *B. fagaroides* (Kunth) Engl. | 1.7% | 14.9% |  |
| *B. glabrifolia* (Kunt) Engl. | 0.4% | 11.1% |  |
| **Convolvulaceae** |  |  |  |
| *Ipomoea murucoides* Roem. & Schult. | 2.1% | 5.5% |  |
| *I. pauciflora* M. Martens &Galeotti | 8.2% | 9.6% |  |
| **Euphorbiaceae** |  |  |  |
| *Sapium macrocarpum* Müll. Arg. | 32.2% | 19.0% |  |
| **Fabaceae** |  |  |  |
| *Conzattia multiflora* (B. L. Rob.) Standl. | -- | 6.3% |  |
| *Lysiloma acapulcense* (Kunth) Benth. | 4.3% | 2.4% |  |
| **Fagaceae** |  |  |  |
| *Quercus obtusata* Humboldt & Bonpl. | 6.4% | -- |  |
| **Lamiaceae** |  |  |  |
| *Salvia sessei* Benth. | 4.7% | -- |  |
| **Malpighiaceae** |  |  |  |
| *Bunchosia canescens* (W. T. Aiton) DC. | -- | 4.3% |  |
| **Meliaceae** |  |  |  |
| *Cedrela odorata* L. | 3.0% | -- |  |
| *Trichilia hirta*L. | 0.4% | 1.9% |  |
| **Oleaceae** |  |  |  |
| *Fraxinus uhdei* (Wenz.) Lingelsh. | 3.9% | -- |  |
| **Rutaceae** |  |  |  |
| *Zanthoxylum fagara* (L.) Sarg. | 3.0% | -- |  |
| **Tiliaceae** |  |  |  |
| *Heliocarpus terebinthinaceus* Hochr. | 0.9% | 1.9% |  |
| Other species | 20.6% | 13.2% |  |

**Table S2**. Bromeliaceae species abundance in two subunits of tropical dry forest in San Andrés de la Cal, Tepoztlán, Mexico. We show the percentage of individuals per species (Vergara-Torres *et al.* 2010, Cortés-Anzures 2015).

| **Species** | **Forest subunit** | |
| --- | --- | --- |
|  | **Lava-rock** | **Limestone-rock** |
| *Tillandsia achyrostachys* E. Morren ex Baker | 12.4% | -- |
| *T. caput-medusae* E. Morren | 2.5% | 2.0% |
| *T. cryptantha* Baker | -- | 0.2% |
| *T. circinnatioides* Matuda | 0.2% | -- |
| *T. hubertiana* Matuda | 2.7% | 20.0% |
| *T. ionantha* Planch | 0.1% | 0.1% |
| *T. makoyana* Baker | 0.2% | 0.2% |
| *T. recurvata* (L.) L. | 76.7% | 2.5% |
| *T. schiedeana* Steud. | 2.4% | 75.0% |
| *Viridantha atroviridipetala* (Matuda) Espejo | 2.8% | -- |

# LITERATURE CITED

Cortés-Anzures BO. 2015. *Distribución de epífitas entre sus forófitos y efecto de Quercus obtusata y Sapium macrocarpum sobre la germinación de Encyclia spatella y Guarianthe aurantiaca en el bosque tropical caducifolio de Tepoztlán*. Master’s Thesis, Universidad Autónoma del Estado de Morelos, México.

Vergara-Torres CA, Pacheco-Álvarez MC, Flores-Palacios F. 2010. Host preference and host limitation of vascular epiphytes in a tropical dry forest of central Mexico. *Journal of Tropical Ecology* **26**: 563–570.
